# Supplementary material for: Comparison of the clinical performance and usefulness of five SARS-CoV-2 antibody tests
Source: PLoS One. 2021 Feb 8;16(2):e0246536. doi: 10.1371/journal.pone.0246536 (PMC7870088; doi:10.1371/journal.pone.0246536)
Supplement: Attachment — Submitted filename: Answer for Comments_Antibody kits R1_V2.0_01182021.docx [file pone.0246536.s001.docx]

**Answers for the comments by the editor and reviewer**

**Comments by the editor:** Thank you for submitting your manuscript to PLOS ONE. After careful consideration, we feel that it has merit but does not fully meet PLOS ONE’s publication criteria as it currently stands. Therefore, we invite you to submit a revised version of the manuscript that addresses the points raised during the review process.
**As stated many report have explored these test, a short update of the most recent litterature in the discussion conclusion deserved to be added in addition to the minor comment from the reviewer.**

**Answer:** Thank you for the editor’s constructive comments to improve our manuscript. We cited the papers published in December 2020 and modified the texts accordingly (Page 3, ll. 55-60; Page 12-14, ll. 236-266, 267).

**Comments by Reviewer #1:**

This is a study that evaluates the Roche anti-SARS-CoV-2 assay and 4 immunocromatographic assays during a short post-infection time. The study is not novel nor unique, but in the pandemic era every study dealing with findings on diagnostics of COVID-19 is valuable. My recommendation is minor revision:
- Page 5/6 The characteristics of the Roche assay are repeated, please merge this to avoid repetitions

**Answer:** Thank you for reviewing our manuscript and providing positive response. We modified the texts accordingly.
